# Effect of Ursodeoxycholic Acid Alone and Ursodeoxycholic Acid Plus Domperidone on Radiolucent Gallstones and Gallbladder Contractility in Humans

**DOI:** 10.1155/2012/159438

**Published:** 2012-05-07

**Authors:** Ilyas Tuncer, Mustafa Harman, Yasar Colak, Ismail Arslan, M. Kursad Turkdogan

**Affiliations:** ^1^Department of Gastroenterology, Istanbul Medeniyet University, Medical Faculty, Goztepe Education and Research Hospital, Istanbul, Turkey; ^2^Departments of Radiology, Yuzuncu Yil University Medical Faculty, Van, Turkey; ^3^Department of Gastroenterology, Bezmiâlem Vakif University Medical Faculty, Istanbul, Turkey

## Abstract

*Background/Aims*. The aim of this study was to compare the effects of ursodeoxycholic acid (UDCA) alone and UDCA plus domperidone on dissolution of solitary or multiple gallstones. *Methods*. Fifty-three patients with cholesterol gallstones were randomized into three treatment groups: group I (*n* = 22) was given UDCA (15 mg/kg/day) alone and group II (*n* = 18) was treated with domperidone (30 mg/day) in addition to UDCA. The control group (*n* = 13) was followed without a medical treatment. Gallbladder volumes and ejection fractions were measured sonographically in all patients before and after treatment. *Results*. After 12 months of treatment, stone dissolution was found in 9 (40.9%) of the patients in group I and 7 (38.8%) of the patients in group II. The difference was statistically significant compared to controls in both treatment groups (*P* < 0.05) but the two groups did not show a difference between each other (*P* > 0.05). All the patients that achieved dissolution had multiple gallstones except for one patient with a solitary stone in group I. Neither monotherapy of UDCA nor the combination with domperidone affected the ejection fraction of gallbladder. *Conclusions*. Combination with domperidone did not potentiate the efficacy of UDCA. It has been observed that both UDCA alone and UDCA plus domperidone treatment did not affect ejection fraction of gallbladder.

## 1. Introduction

Gallstones are a major cause of morbidity and mortality throughout the world. The prevalence of gallstone disease in western industrialized nations is about 10–15% [1–3]. Although, 2/3 of all gallstones remain asymptomatic (so called silent gallstone), recurrent episodes of upper abdominal pain related to gallstones are the most common indication for the treatment of gallstones [2, 4]. Cholecystectomy is the standard and definitive treatment for symptomatic gallbladder stones and can be performed regardless of the type, number, and size of the stones [1, 5, 6]. However, some, 20%, of patients continue to suffer from pain after cholecystectomy [7].

Nonsurgical management of gallstones has made considerable progress within the past 20 years [2, 8]. Recent studies have raised the possibility that cholesterol-lowering agents that inhibit hepatic cholesterol synthesis (statins) or intestinal cholesterol absorption (ezetimibe), or drugs acting on specific nuclear receptors involved in cholesterol and bile acid homeostasis, may offer, alone or in combination, additional medical therapeutic tools for treating cholesterol gallstones [3, 5, 6]. Ursodeoxycholic acid is a bile salt that reduces the secretion of cholesterol into bile and increases cholesterol solubility. It may also improve gallbladder emptying [2, 4, 9]. Oral litholysis with UDCA induces cholesterol desaturation of bile and may lead to gallstone dissolution in patients with small, radiolucent, cholesterol-enriched stones in a functioning gallbladder with a patent cystic duct [5, 6, 10]. It has been suggested that treatment with bile acids may inhibit gallstone related-symptoms and complications even in patients in whom gallstone dissolution is incomplete [4, 7, 10].

 The motility of the gallbladder is another key player in gallstone pathogenesis because enlarged fasting gallbladder volume, together with impaired postprandial and interdigestive gallbladder emptying, is frequent and distinctive features in gallstone patients [5]. Impaired gallbladder motility in patients with gallstone disease can be corrected by prokinetic drugs [11, 12]. Domperidone, a peripheral dopamine 2-receptor antagonist, regulates the motility of gallbladder, gastric, and small intestinal smooth muscle. It is rapidly absorbed after oral administration, and few side effects have been reported [12–14].

The aim of this study was to investigate the effects of UDCA-alone and UDCA plus domperidone on stone dissolution, fasting gallbladder volume, and gallbladder ejection fraction in solitary and multiple radiolucent gallbladder stones.

## 2. Methods

Seventy-five patients (64 female, 11 male; mean age: 45.5 ± 11; age range: 25–77) with asymptomatic or mild symptomatic cholesterol gallstone were enrolled in this randomized study.

Evaluation for each subject included a complete history and physical examination, complete blood count, serum cholesterol, triglycerides, aminotransferases, total bilirubin and alkaline phosphatase, serum amylase, abdominal X-rays, and abdominal ultrasound.

All gallstone patients were candidates for oral bile acid therapy (radiolucent gallstones, less than 2 cm in diameter, good gallbladder emptying, patent cystic duct, mild symptoms, and no evidence of acute cholecystitis). Fifty-eight patients had two or more gallbladder stones termed as multiple stones whereas remaining 17 patients had solitary gallbladder stones. There was no history of recent pancreatitis, cholangitis, and biliary colic in any of the patients. Patients who had a history of systemic disease, current pregnancy or lactation, alcohol, and medication were excluded.

After 12 hours overnight fast, fastening, and postprandial gallbladder volumes were measured sonographically before and 60 minutes after ingestion of a standard liquid test meal (18.4 g fat, 16 g protein, 240 kcal per 150 mL) and postprandial ejection fractions were calculated in all patients. Gallbladder volume was measured using a real-time ultrasound scanner (Toshiba SSA-270A, Japan, with a 3.75-mHz curved transducer). Sonographic studies were performed by the same investigator. Gallbladder dimension in the longitudinal, transvers, and saggittal planes were obtained. The smallest volume obtained after feeding at 60th minute was determined as postprandial (or residual) gallbladder volume. Gallbladder volume and ejection fraction were determined using the following formulas [15]:

(1) $$\text{Gallbladder}\;\text{volume}\;\left(V\right)=\frac{\pi }{6}\times \left(L\times W\times H\right)$$

*V* = gallbladder volume, *L* = saggittal length, *W* = width, *H* = axial height,

(2) $$\text{Ejection}\;\text{fraction}\left(E\right)=\frac{\left(V_{0}-V_{t}\right)}{V_{0}}\times 100$$

*E* = ejection fraction, *V*_0_ = fasting gallbladder volume, *V*_*t*_ = postprandial (residual) gallbladder volume at time 60 minute.

All patients gave written informed consent and the study was approved by the local research ethics committee. The patients were randomized into three treatment groups: group I (*n* = 30) received UDCA alone (15 mg/kg/day, Ursofalk, Falk Pharma GmbH, Germany); group II (*n* = 30) received UDCA plus 30 mg/day domperidone (Motilium, Janssen-Cilag, Turkey). The control group (*n* = 15) was followed without a medical treatment.

The physical examination and blood samples were repeated in each patient in 3-month intervals during the treatment period. In addition, gallbladder motility and gallstone dissolution rates at the third, sixth, ninth and the twelfth months were evaluated by ultrasonography in all patients.

Patients were not allowed to take any other drugs affecting biliary lipids or cholesterol biosynthesis. Patients were excluded from the study if they exhibited any abnormality of gallbladder, bile duct, or pancreatic function as determined by X-ray or ultrasonography. The treatments were discontinued at the 6th month if stone dissolution was not achieved.

 Results are presented as mean values ± standard deviation (x ± SD). For statistical comparison of the three groups before and after treatment one-way ANOVA variance analysis was used. Within group analysis for comparison before and after treatments was performed with paired-student's *t*-test. Stone dissolution rates were compared with chi-square test. The difference was considered statistically significant if the *P* value was less than 0.05.

## 3. Results

Six patients in group I, nine patients in group II were dropped out of the study because of nonattendance or incompatibility with the treatments. In addition, 2 patients in group I were excluded from the study due to severe biliary colic in one and acute edematous pancreatitis in the other. Three patients in group II and two patients in group III underwent urgent laparoscopic cholecystectomy and therefore excluded. The final analysis was performed with 22 patients (18 female, 4 male) from the UDCA group, 18 patients (16 female, 2 male) from UDCA plus domperidone group, and 13 patients (11 female, 2 male) from the control group. There were no statistically significant differences between groups regarding demographic, clinical and laboratory data on admission. The patient characteristics are presented in Table 1.

**Figure 1:**
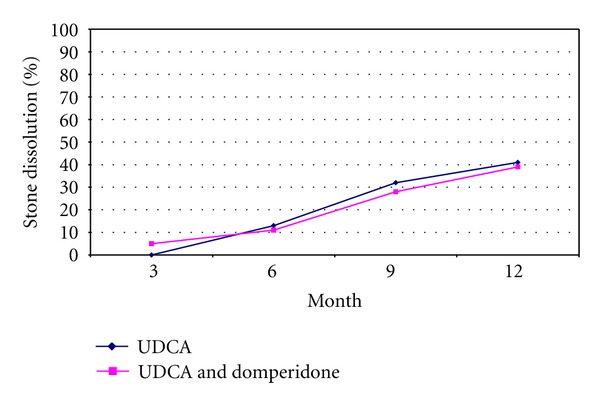
Stone dissolution rates with time in both treatment groups.

At the end of the treatment period of 12 months, complete stone clearance was achieved in 9 (40.9%) of the patients in UDCA group and in 7 (38.8%) of the patients in UDCA plus domperidone. The difference was statistically significant compared to controls for both treatment groups (*P* < 0.05) but the two groups did not show a difference between each other (*P* > 0.05). Of the 16 cases that achieved dissolution, all had multiple gallstones except for one in UDCA group. Solitary gallstones did not disappear in any subject treated with UDCA plus domperidone combination (Figure 1 and Table 2). The gallstone dissolution rate at 12 months was not higher for UDCA plus domperidone than treatment with UDCA-alone. Gallstones were not dissolved in any subject in control group.

At the end of the treatment period, no significant differences in terms of fasting and postprandial gallbladder volumes were observed between the groups. Similarly, gallbladder ejection fraction before and after the end of treatment was similar for all three groups (*P* > 0.05) (Table 3).

Laboratory tests including serum alkaline phosphatase, cholesterol, aspartate, and alanine aminotransferase were measured monthly, and results of these tests showed no change during the treatment periods in all groups (Table 3).

Both regimens showed no significant difference in terms of side effects or drop-out rate. UDCA and domperidone were generally tolerated quite well. The drugs did not cause any severe gastrointestinal or other side effects that required hospitalisation. The only significant side effect was a mild-to-moderate upper-right quadrant pain sometimes necessitating spasmolytics without any laboratory abnormality in 4 cases in UDCA group and 2 cases in the combination group, all of which had successful stone dissolution. There was a remarkable decrease in symptoms in patients that achieved stone dissolution. In patients with partial stone dissolution UDCA therapy was continued beyond the study period.

## 4. Discussion

Cholelithiasis is one of the most common and costly digestive diseases. Gallstones are usually asymptomatic and no treatment is generally required and laparoscopic cholecystectomy is first-line therapy for symptomatic gallstones [6, 7]. So far, UDCA has been recommended as first-line pharmacological therapy in a subgroup of symptomatic patients with small, radiolucent cholesterol gallstones in a functioning gallbladder with a patent cystic duct, and its long-term administration has been shown to promote the dissolution of cholesterol gallstones [3, 5, 6]. Therapy with bile salts is suitable for only a minority of patients with symptomatic cholesterol gallstones who refuse surgery or have an increased surgical risk [2, 10, 16]. UDCA reduces the synthesis of cholesterol in the liver and its secretion into bile. UDCA also prolongs the nucleation time of gallbladder bile by shifting cholesterol from vesicles to micelles [2, 10, 17].

The therapeutic success of the medical dissolution of gallstones depends substantially on the following factors: (i) patient selection, (ii) dosage of bile acid, and (iii) therapy duration. The ideal patient to be treated by medical therapy is mildly symptomatic, non-obese, with functioning gallbladder and with radiolucent, floating stones, not larger than 5 mm in diameter [10, 18, 19].

In the current study, we achieved complete gallstone dissolution rates of 40.9% with UDCA and 38.8% with combination therapy at 12 months. Stone dissolution rate was higher in patients with multiple stones compared to those with solitary stones but this difference was not statistically significant. Combination of UDCA with domperidone did not improve complete gallstone dissolution rate, gallbladder fasting volume, and gallbladder ejection fraction. In both treatment groups, the use of UDCA reduced incidence of biliary pain. Stone dissolution rates were increased with the lengthening of treatment periods. Younger patients demonstrated higher rates of dissolution compared to elderly subjects. The only solitary stone dissolved with the treatment was 7 mm diameter (see Figure 1, Table 2).

Based on a simple correlation with the gallstone dissolution rate, small size, radiolucency of gallstone, and preserved gallbladder function have been listed as indication criteria for UDCA therapy. Size rather than number of stones is the primary determinant of the dissolution rate [18, 20, 21]. Gallstones smaller than 5 mm show a better dissolution rate than those exceeding 5 mm [20]. Stones of less than 5 mm diameter can be dissolved in almost 79% of cases, whereas the success rates drop to roughly one-third with stones larger than 10 mm in diameter [10, 18]. With stones more than 15–20 mm diameter, the dissolution time is extremely long [10]. Studies using strict inclusion criteria have found dissolution rates of 50 to 60%. However, dissolution rates as low as 23% have been reported in less well-selected populations [9]. UDCA therapy can be useful in patients with biliary sludge or microlithiasis, especially if recently developed [18]. UDCA completely dissolved the stones in 37% of all patients [16]. Jazrawi et al. [22] have found complete stone dissolution as 78% with UDCA treatment at the end of 6 months, whereas Schoenfield and Marks [23] reported a success rate of 50% at the end of 24 months. On the other hand, Bazzoli et al. [24] achieved a stone dissolution rate of as low as 13% at the end of 6 months. The wide variation in the reported response rates can be attributable to differences in patient selection, doses of bile acid, treatment duration, and the diagnostic techniques used to document complete stone dissolution [16].

In previous studies different results have been reported about the effects of UDCA alone or in combination with other drugs. Combination of UDCA with chenodeoxycholic acid therapy enhances the biliary levels of UDCA [25]. Zuin et al. [26] have obtained gallstone dissolution rate of 83% with UDCA plus chenodeoxycholic acid combination in 12 months, whereas Petroni et al. [27] obtained a 30% dissolution rate with the same combination over 24 months. Moreover, Tazuma et al. [28] have found 70% stone dissolution in multiple stones and 25% in solitary stones after 12 months treatment with UDCA. In the same study, combination with simvastatin was not proved superior compared to UDCA treatment alone and was not proved superior for solitary stones. In our study stone dissolution rate was lower than those reported by Tazuma et al. [28]. As in previous studies, we failed to show superiority of combination therapy over UDCA-alone treatment on dissolution of multiple or solitary stones.

Gallbladder emptying abnormalities are common in patients with gallstones, and it has been hypothesized that they contribute to the increased incidence of gallbladder stones [4]. In the treatment of gastrointestinal motility disorders 3 prokinetic agents (metoclopramide, domperidone, and cisapride) have mainly been used. They are differentiated from their pharmacological mode of action, their clinical efficacy, and tolerability. Domperidone is a pure dopamine receptor antagonist. It antagonizes noradrenaline- and dopamine-induced relaxations of smooth muscles [29]. We wondered whether the therapeutic effects of domperidone and UDCA in gallstone disease might be mediated by changes in gallbladder contractility. In our study, we found that neither the UDCA nor domperidone altered fasting gallbladder volume and ejection fraction in patients with gallstone at the end of 12 months. Our findings suggest that the dissolution of gallstones during oral bile acid administration is not paralleled by an improvement in gallbladder function. In the literature, different results have been reported about the effects of prokinetics drugs on gallbladder motility. Tankurt et al. [12] and Nakayama et al. [30] demonstrated that domperidone increases gallbladder contractility in healthy subjects in contrast to Mangiameli et al. [13] who showed that domperidone has no effect on contractility. In studies performed with cisapride, Kapicioglu et al. [31] demonstrated that this drug did not change the fasting mean gallbladder volume as compared to the baseline in the healthy subjects. However, this study showed that the administration of cisapride causes gallbladder volume reduction in diabetic patients. Differently, Gürsoy et al. [32] showed that cisapride did not change the contractility of gallbladder in diabetic patients. Recently proposed some molecular mechanisms bring up alternative treatments for bile stones. For example, the human apical sodium-dependent bile acid transporter (hASBT) plays a critical role in the enterohepatic circulation of bile acids and cholesterol homeostasis whose expression is regulated by FXR [33, 34]. Therefore, the use of FXR agonists could have potential therapeutic applications in hypercholesterolemia and in cholestasis, but not in cholesterol gallstone therapy.

We conclude that UDCA treatment should be preferred for patients with small multiple gallstones and that administration of combined UDCA and domperidone is not likely to provide an effective combination. Gallbladder fasting volume and ejection fraction did not change at the end of gallstone dissolution treatment in radiolucent gallstone patients. We also demonstrate that UDCA and domperidone combination have no effect on gallbladder contractility in patients with gallstone.

In conclusion, we found higher dissolution rates in patients with multiple stones compared to these with solitary stones as in previous studies. Younger patient age and longer treatment periods increased the chance of successful treatment. Long-term medical treatment with bile salts may help to reduce gallstone-related complications and need for surgery especially in multiple gallstones in which both the risk of complication and the benefit of therapy are greatest.

## Figures and Tables

**Table 1:** Demographic and clinic characteristics of groups before the treatment*.

Parameters	UDCA (*n* = 22)	UDCA and domperidone (*n* = 18)	Control (*n* = 13)
Mean age	45.4 ± 12	42.7 ± 13	49.0 ± 8
Gender (F/M)	18/4	16/2	11/2
BMI (kg/m^2^)	29.0 ± 4	27.1 ± 3	30.3 ± 5
Glucose (mg/dL)	90.5 ± 11	99.1 ± 20	96.2 ± 7
Cholesterol (mg/dL)	182.4 ± 34	186.1 ± 30	177.0 ± 26
HDL-cholesterol (mg/dL)	49.7 ± 16	53.5 ± 17	43.0 ± 9
LDL-cholesterol (mg/dL)	105.0 ± 35	104.3 ± 51	108.4 ± 15
Triglyceride (mg/dL)	136.5 ± 75	157.4 ± 90	145.7 ± 81
Solitary stone	6	4	2
Diameters of solitary stones (mm)	8.1 ± 3.1	8.2 ± 3.3	8 ± 3.6
Multiple stone	16	14	11
FGV_av_ (mL)	26.6 ± 9.6	27.8 ± 13.4	25.4 ± 6.2
RGV_av_ (mL)	7.0 ± 3	7.8 ± 5	6.3 ± 6
EF_av_ (%)	73.6 ± 15	71.9 ± 19	75.1 ± 18

*Results are expressed as mean ± SD.

BMI: body mass index; FGV_av_: average fasting gallbladder volume; RGV_av_: average residual gallbladder volume; EF_av_: average ejection fraction.

**Table 2:** Characteristics of the patients of whom stone dissolution was achieved or not*.

Parameters	UDCA alone				UDCA and domperidone			
	Solitary (*n* = 6)		Multiple (*n* = 16)		Solitary (*n* = 4)		Multiple (*n* = 14)	
	SD^Φ^ (+)*n* = 1	SD (−)*n* = 5	SD (+)*n* = 8	SD (−)*n* = 8	SD (+)*n* = 0	SD (−)*n* = 4	SD (+)*n* = 7	SD (−)*n* = 7
Age (mean)	35	48.2 ± 8	35.7 ± 11	46.9 ± 13	—	45.2 ± 9	39.4 ± 5	44.6 ± 12
Gender (F/M)	1/—	5/—	7/2	5/2	—	4/—	6/1	5/2
BMI (kg/m^2^)	28	31	28.5	29	—	28.5	27	27
SS_av_ (mm)	7	13	—	—	—	14.8	—	—
FGV_av_ (mL)	23.6	25.4 ± 7	27.9 ± 8	26.6 ± 8	—	24.6 ± 9	26.8 ± 4	29.2 ± 17
EF_av_ (%)	69.7	72.0 ± 12	75.8 ± 16	75.5 ± 17	—	74.6 ± 10	72.3 ± 13	76.4 ± 21

*Results are expressed as mean ± SD.

^Φ^SD: stone dissolution; BMI: body mass index; SS_av_: average stone size; FGV_av_: average fasting gallbladder volume; EF_av_: average gallbladder ejection fraction.

**Table 3:** Characterisitcs of patients in treatment groups in pre- and posttreatment*.

Parameters	UDCA alone			UDCA and domperidone		
	Pretreatment	Posttreatment	*P*	Pretreatment	Posttreatment	*P*
BMI (kg/m^2^)	29.0 ± 4	28.8 ± 6	NS	27.1 ± 3	27.1 ± 5	NS
Glucose (mg/dL)	90.5 ± 11	96 ± 6	NS	99.1 ± 20	88 ± 13	NS
Cholesterol (mg/dL)	182.4 ± 34	178 ± 36	NS	186.1 ± 30	178 ± 59	NS
HDL-Cholesterol (mg/dL)	49.7 ± 16	51.2 ± 9	NS	53.5 ± 17	56.7 ± 10.6	NS
LDL-Cholesterol (mg/dL)	105.0 ± 35	105 ± 42	NS	104.3 ± 51	101 ± 56	NS
Triglyceride (mg/dL)	136.5 ± 75	142 ± 52	NS	157.4 ± 90	178 ± 49	NS
Solitary stone	6	5	NS	4	4	NS
Multiple stone	16	8	NS	14	7	NS
FGV_av_ (mL)	26.6 ± 9.6	27.0 ± 5.2	NS	27.8 ± 13.4	29.2 ± 6.4	NS
RGV_av_ (mL)	7.0 ± 3	6.5 ± 2.6	NS	7.8 ± 5	7.8 ± 3.7	NS
EF_av_ (%)	73.6 ± 15	75.9 ± 16	NS	71.9 ± 19	73.2 ± 14	NS

*Results are expressed as mean ± SD.

BMI: body mass index; FGV_av_: average fasting gallbladder volume; RGV_av_: average residual gallbladder volume; EF_av_: average ejection fraction.

NS: not significant.
